# Supplementary material for: Preparation of Gum Arabic–Maltose–Pea Protein Isolate Complexes for 1−Octacosanol Microcapsule: Improved Storage Stability, Sustained Release in the Gastrointestinal Tract, and Its Effect on the Lipid Metabolism of High−Fat−Diet−Induced Obesity Mice
Source: Foods. 2022 Dec 26;12(1):112. doi: 10.3390/foods12010112 (PMC9818909; doi:10.3390/foods12010112)
Supplement: Supplementary file 1 [file foods-12-00112-s001.zip › foods-1947468-supplementary.pdf]

Supplementary Table S1 The configuration of stock solution in simulated gastrointestinal tract

	Stock solution volume (mL)	Storage solution concentration (g/L)
KCl	250	37.3
KH_2PO_4	250	68
$NaHCO_3$	250	84
NaCl	250	117
$MgCl_2(H2O)_6$	100	30.5
$(NH_4)_2CO_3$	100	48
$CaCl_2(H_2O)_2$	100	43.8 (0.3 mol/L)

Supplementary Table S2 The components of SGF and SIF[a]

Form	Stock conc. (mol/L)	SGF (pH = 3)		SIF (pH = 7)	
		Vol. of stock (mL)	Conc. in SGF (mmol/L)	Vol. of stock (mL)	Conc. in SIF (mmol/L)
KCl	0.50	17.50	6.90	13.60	6.80
KH_2PO_4	0.50	2.25	0.90	1.60	0.80
$NaHCO_3$	1.00	31.25	25.00	85.00	85.00
NaCl	2.00	29.50	47.20	19.20	38.40
$MgCl_2(H2O)_6$	0.15	1.00	0.10	2.20	0.33
$(NH_4)_2CO_3$	0.50	1.25	0.50	-	-
$CaCl_2(H_2O)_2$[b]	0.30	-	1.50	-	0.60

a) The composition and configuration of the simulated digestion solution were shown in the international convention, and the final constant volume was 1000 mL.

b) In order to prevent calcium precipitation, $CaCl_2(H_2O)_2$ was added before *in vitro* digestion.

Supplementary Table S3 The primer's sequences for the genes

Gene	Forward (5′-3′)	Reverse (5′-3′)
GK	ATGTCGCTTTCCAACAAGCTG	GCTCCATTGTCCAAGCAGAAT
Serbp1c	GCAGCCACCATCTAGCCTG	CAGCAGTGAGTCTGCCTTGAT
Fas	ATGCTGTGGATCTGGGCTGTC	CAGTTTCACGAACCCGCCTC
Acc1	GCACGTTCGATAGCACACCAC	ATGGCATACATACCAATCTCTCTA
β-actin	TGTCACCAACTGGGACGATA	GGGGTGTTGAAGGTCTCAAA
